# Mast Cell Mediated Regulation of Small Intestinal Chloride Malabsorption in SAMP1/YitFc Mouse Model of Spontaneous Chronic Ileitis

**DOI:** 10.3390/cells10030697

**Published:** 2021-03-21

**Authors:** M Motiur Rahman, Sheuli Afroz, Subha Arthur, Uma Sundaram

**Affiliations:** Department of Clinical and Translational Sciences and Appalachian Clinical and Translational Science Institute, Joan C. Edwards School of Medicine, Marshall University, 1600 Medical Center Drive, Huntington, WV 25701, USA; rahmanmd@marshall.edu (M.M.R.); afroz@marshall.edu (S.A.); arthursu@marshall.edu (S.A.)

**Keywords:** SAMP1/YitFcs, down regulated in adenoma, DRA, inflammatory bowel disease, Crohn’s disease, mast cells, ketotifen

## Abstract

In Inflammatory Bowel Disease (IBD), malabsorption of electrolytes (NaCl) results in diarrhea. Inhibition of coupled NaCl absorption, mediated by the dual operation of Na:H and Cl:HCO_3_ exchangers on the brush border membrane (BBM) of the intestinal villus cells has been reported in IBD. In the SAMP1/YitFcs (SAMP1) mice model of spontaneous ileitis, representing Crohn’s disease, DRA (Downregulated in Adenoma) mediated Cl:HCO_3_ exchange was shown to be inhibited secondary to diminished affinity of the exchanger for Cl. However, NHE3 mediated Na:H exchange remained unaffected. Mast cells and their secreted mediators are known to be increased in the IBD mucosa and can affect intestinal electrolyte absorption. However, how mast cell mediators may regulate Cl:HCO_3_ exchange in SAMP1 mice is unknown. Therefore, the aim of this study was to determine the effect of mast cell mediators on the downregulation of DRA in SAMP1 mice. Mast cell numbers and their degranulation marker enzyme (β-hexosaminidase) levels were significantly increased in SAMP1 mice compared to control AKR mice. However, treatment of SAMP1 mice with a mast cell stabilizer, ketotifen, restored the β-hexosaminidase enzyme levels to normal in the intestine, demonstrating stabilization of mast cells by ketotifen. Moreover, downregulation of Cl:HCO_3_ exchange activity was restored in ketotifen treated SAMP1 mice. Kinetic studies showed that ketotifen restored the altered affinity of Cl:HCO_3_ exchange in SAMP1 mice villus cells thus reinstating its activity to normal. Further, RT-qPCR, Western blot and immunofluorescence studies showed that the expression levels of DRA mRNA and BBM protein, respectively remained unaltered in all experimental conditions, supporting the kinetic data. Thus, inhibition of Cl:HCO_3_ exchange resulting in chloride malabsorption leading to diarrhea in IBD is likely mediated by mast cell mediators.

## 1. Introduction

Crohn’s disease (CD), part of a group of gastrointestinal disorders named as inflammatory bowel diseases (IBD), is characterized by transmural inflammation and skip lesions that might occur throughout the length of the gastrointestinal tract [1]. The incidence of CD, although variable in different geographical regions, has been steadily increasing over the years in North America, Western Europe, Asia, and South America [2,3], particularly in the United States. The etiopathogenesis of this incurable disease is said to be complex and is known to involve interacting elements including genetic susceptibility of the host, intestinal microbiota, environmental factors, and immunological abnormalities of the host [4]. The chronic inflammation of CD is due to dysregulated host immune inflammatory response leading to its pathogenesis [5]. Most importantly, many of the immune inflammatory mediators released in the chronically inflamed mucosa of IBD profoundly affect the absorptive and secretory properties of intestinal epithelial cells leading to diarrhea, malnutrition, and weight loss in IBD patients [6,7]. More specifically, as demonstrated by multiple studies, impaired intestinal absorption of nutrients (glucose, amino acids, vitamins etc.) and electrolytes (Na, Cl etc.) have been shown to be caused by specific immune-inflammatory meditators affecting epithelial transport properties [8,9,10,11,12].

In the mammalian small intestine, electroneutral NaCl absorption is the predominant mechanism of absorption of the electrolytes Na and Cl, which occurs through the functional coupling of brush border membrane (BBM) exchangers mediating Na:H exchange (NHE3/SLC9A3) and Cl:HCO_3_ exchange (DRA, Downregulated in Adenoma/SLC26A3 and PAT1, Putative Anion Transporter-1/SLC26A6) [13,14,15,16]. Impairment of this electroneutral exchange is believed to be the prime cause of IBD associated diarrhea [17,18]. In addition, considerable evidence found in the literature suggests the direct role of pro-inflammatory cytokines and eicosanoids on the dysregulation of electrolyte absorption in IBD [13,18,19]. Another key mucosal immune component that might have a regulatory role on IBD associated malabsorption of electrolytes is the mast cell. Activated mast cells are known to release an array of immune mediators that affect the mucosal barrier function including histamine, 5-hydroxytryptamine (5-HT), neutral proteases (tryptases, chymases and carboxypeptidase A), prostaglandins, leukotrienes, platelet activating factor, and a variety of cytokines including TNFα, IL-3, IL-4, IL-5, etc. [20,21,22,23]. Moreover, many of these mast cell immune mediators have been implicated in altered epithelial ion transport processes [24,25].

SAMP1/YitFcs (SAMP1) is a recombinant-inbred mice model that spontaneously develops ileitis, therefore, unlike other animal models, does not require any biological or chemical agent to induce inflammation [26]. The characteristics of SAMP1 ileitis is very similar in many features to the human IBD such as the presence of mixed inflammatory infiltrate and epithelial injury including villus shortening etc. and hence is a highly appropriate in vivo model to study small intestinal inflammation. In this animal model, we have previously demonstrated that Cl:HCO_3_ exchange was downregulated in ileal villus cell BBM. Moreover, this downregulation of Cl:HCO_3_ exchange was found to be secondary to the altered affinity of the Cl:HCO_3_ exchanger for chloride [27]. Furthermore, molecular studies demonstrated that only DRA, but not PAT1, had altered phosphorylation levels at its serine and threonine residues, which likely mediated its altered affinity for chloride [28]. These data indicated that DRA, rather than PAT1, is the anion exchanger responsible for chloride malabsorption during chronic ileitis. With regard to Na:H exchange in the same study, Na:H exchange mediated by NHE3 remained unaffected in the BBM of villus cells [27], which led us to believe that dysregulated traditional NaCl coupled absorption may not be the viable reason for Na and Cl malabsorption leading to diarrhea in the SAMP1 model of chronic ileitis. Given this background and the importance of DRA in the IBD associated malabsorption of chloride, we determined if mast cell mediators could be responsible for the specific downregulation of DRA mediated chloride malabsorption in the SAMP1 model of spontaneous ileitis. Thus the aim of the current study was to determine how mast cell stabilization may affect the downregulation of DRA in the BBM of villus cells in the SAMP1 mice model of chronic ileitis and to define the functional and molecular aspects of DRA regulation by mast cells.

## 2. Materials and Methods

### 2.1. Animal Models and Drug Treatment

AKR/J mice (males) and SAMP1/YitFcs mice (males) were obtained from The Jackson Laboratory (Bar Harbor, ME, USA) and were used in the current study at 10 weeks of age. The animals were maintained in a 12-h light/dark cycle with free access to food and water. Mice that were injected intraperitoneally with distilled water were used as untreated controls. For drug treatment, AKR and SAMP1/YitFcs mice were intraperitoneally injected for 2 days with ketotifen (10 mg/kg body weight), a noncompetitive H1-antihistamine and mast cell stabilizer. Ketotifen was obtained from Cayman Chemicals, Ann Arbor, MI, USA. All the components of this animal study including animal handling procedures, treatments and euthanization were approved by Marshall University’s Institutional Animal Care and Use Committee (Protocol Reference number 743).

### 2.2. Cell Isolation

Ca^++^ chelation technique was used for the isolation of small intestinal villus cells from treated and untreated AKR and SAMP1/YitFcs mice as described before [29,30]. Small intestinal villus cells were also obtained from treated and un-treated mice by scrapping the intestinal mucosa. The cells were flash frozen immediately in liquid nitrogen and stored at  −80 °C until experimental use.

### 2.3. β-Hexosaminidase Activity Assay

β-hexosaminidase activity assay was performed to detect mast cell degranulation levels in vivo. In brief, homogenized villus cells from different experimental conditions were centrifuged at 14,000× *g* for 10 min at 4 °C and the supernatant was used for the assay. β-hexosaminidase activity was then measured in an equal amount of protein using GSI Beta-N-acetylhexosaminidase colorometric assay kit (Cat. GR107044, Genorise Scientific, Inc., Glen Mills, PA, USA). The results were expressed as percentage of β-hexosaminidase activity relative to control.

### 2.4. BBM Vesicles (BBMV) Preparation

Mg^++^ chelation and differential centrifugation techniques were used for the ileal villus BBM vesicles (BBMV) preparation as previously reported [13,30]. Villus BBMV was suspended in an appropriate vesicle medium for uptake experiments. For Western blot studies, villus BBMV was suspended in an appropriate protein extraction buffer.

### 2.5. ^36^Cl^−^ Uptake Studies to Determine Cl^−^/HCO_3_^−^ Exchange in BBMV

The rapid-filtration technique was employed for the ^36^Cl^−^ uptake studies in BBMVs. Cl^−^/HCO_3_^−^ exchange experiments were performed by resuspending BBMV in vesicle medium containing 105 mM N-methyl-D-glucamine (NMG) gluconate, 50 mM HEPES-Tris pH 7.5 with either 50 mM KHCO_3_^−^ gassed with 5% CO_2_ + 95% N_2_ or 50 mM potassium gluconate gassed with 100% N_2_. The reaction was started by adding 5 μL of vesicle to 95 μL reaction medium containing 5 mM NMG ^36^Cl^−^, 149.7 mM potassium gluconate, 50 mM MES-Tris pH 5.5 and either 0.3 mM KHCO_3_ gassed with 5% CO_2_, 95% N_2_ or 150 mM potassium gluconate gassed with 100% N_2_. One mM 4,4-diisothiocyanatostilbene-2,2-disulfonic acid disodium salt (DIDS), a potent anion exchange inhibitor, was used as the inhibitor. The uptake was stopped at the desired time with ice cold stop solution containing 50 mM HEPES-Tris buffer (pH 7.5), 0.10 mM MgSO_4_, 50 mM potassium gluconate, and 100 mM NMG gluconate. The mixture was filtered on 0.45 μm Millipore (HAWP) filters and washed twice with 5 mL ice-cold stop solution. Filters with BBMV were dissolved in 4 mL scintillation fluid (Ecoscint, National Diagnostics), and radioactivity was determined in a Beckman 6500 Beta Scintillation Counter. Results were calculated as the HCO_3_ dependent DIDS sensitive Cl^−^ uptake.

### 2.6. ^22^Na Uptake Studies to Determine Na/H Exchange in BBMV

NHE3 activity was measured as pH dependent and amiloride sensitive ^22^Na uptake. ^22^Na uptake was measured in BBMV by the rapid filtration technique as previously described [31,32]. Briefly, 5 μL of BBMV was suspended in vesicle medium and incubated in 95 μL of reaction medium and with or without 1 mM amiloride. At 60 s, the uptake was arrested by mixing with ice-cold stop solution and processed as described for ^36^Cl^−^ uptake studies in BBMV.

### 2.7. Cl^−^/HCO_3_^−^ Exchange Kinetic Studies in Intact Villus Cells

For Cl^−^/HCO_3_^−^ exchange kinetic studies, ^36^Cl^−^ uptake was performed in isolated intact villus cells. Briefly, intact villus cells (100 mg wet wt.) were resuspended in either 5 mM of N-methyl-D-glucamine (NMG), 50 mM of KHCO_3,_ and 50 mM of HEPES-Tris pH 7.5 or 5 mM of NMG, 50 mM of potassium gluconate and 50 mM of HEPES-Tris pH 7.5. Ten µL of villus cells were then incubated in 90 µL of appropriate reaction medium that contained varying concentrations of NMG ^36^Cl^−^ (0.5, 1, 5, 10, 25, 50 mM) for 30 s. The mixture was then filtered on 0.65 μm Millipore (Bedford, MA, USA) filters and washed twice with ice cold-stop solution. The filter was dissolved in 4 mL Ecoscint solution and the radioactivity was determined in a Beckman Coulter LS6500 Scintillation counter. Uptake values were analyzed for simple Michaelis–Menten kinetics using a non-linear regression data analysis using GraphPad Prism 8 (San Diego, CA, USA).

### 2.8. Real Time qPCR Analysis

Total RNA was extracted from treated and untreated mice small intestinal villus cells using RNeasy mini kit (74106; Qiagen, Germantown, MD, USA). RTQ-PCR was performed by a two-step method. First strand cDNA synthesis was performed with High capacity cDNA Reverse Transcription kit (4368814; Applied Biosystems, Foster City, CA, USA). The cDNAs generated were used as templates for RTQ-PCR. The reactions were performed using TaqMan universal PCR master mix from Applied Biosystems on an Applied Biosystems Step One Plus Real-time PCR system. RTQ-PCR for mouse specific DRA (Mm00445313 m1) was performed using TaqMan^®^ Gene Expression Assays obtained from Applied Biosystems. Mouse specific β-actin (Mm01205647 g1) RT-qPCR, which served as the endogenous control, was run along with the DRA in similar experimental conditions (40 cycles: 95 °C for 15 s and 60 °C for 1 min). The data obtained with β-actin was used to normalize the expression levels of DRA between individual samples. RT-qPCR experiments were performed in triplicate using total RNA extracted from isolated villus cells.

### 2.9. Western Blot Analysis

BBM protein extract from isolated small intestine villus cells solubilized in RIPA buffer (50 mM Tris-HCl pH 7.4, 1% Igepal, 150 mM NaCl, 1 mM EDTA, 1 mM PMSF, 1 mM Na_3_VO_4_, 1 mM NaF) with protease inhibitor cocktail (SAFC Biosciences, Lenexa, KS, USA) was used for the Western blot analyses. The equal amount of proteins from different experimental conditions were mixed with sample buffer (100 mM Tris, 25% glycerol, 2% SDS, 0.01% bromophenol blue, 10% 2-ME, pH 6.8) and denatured. Proteins were separated by electrophoresis on an 8% polyacrylamide gel. The separated proteins were transferred to a BioTrace PVDF membrane which was blocked with 5% nonfat dried milk in TBS (20 mM Tris, pH 7.5, 150 mM NaCl) with 0.1% Tween-20 and probed with anti-DRA antibody (diluted 1:1000) (sc376187, Santa Cruz Biotechnology, Inc., Dallas, TX, USA) at 4 °C for overnight. Western blot analysis of Ezrin protein, detected with anti-Ezrin (diluted 1:1000) (MAB3822-C, Millipore, Temecula, CA, USA), was used to confirm equal protein loading. The primary antibody bound to the DRA and Ezrin proteins were detected with horseradish peroxidase coupled secondary antibody (diluted 1:1000) (1706516, Bio-Rad Laboratories, Life Science Group, Hercules, CA, USA) for an hour at room temperature. The blots were then developed with an enhanced chemiluminescent detection reagent (GE Healthcare, Chicago, IL, USA). The chemiluminescence was detected using a FluorChem M instrument (Alpha Innotech, San Leandro, CA, USA) and the DRA specific protein density was then calculated with ImageJ PC-based software (National Institute of Health).

### 2.10. Histology and Immunofluorescence Study

A small portion of ileum was fixed in 10% neutral buffered formalin and processed for paraffin embedding. Paraffin sections (5 μm) were cut for use in histology, and immunofluorescence. To visualize the mast cells, sections were dewaxed with two washes in xylene and hydrated by serial passage through graded alcohols. Sections were stained with toluidine blue, a metachromatic dye for mast cells, made up of 0.1% toluidine blue O (Sigma-Aldrich Corporation, St. Louis, MO, USA) in 1% sodium chloride at pH 2.3 for 3 min. Sections were washed in distilled water, dehydrated, and mounted. Images were visualized in a light microscope and captured at different magnification. Mast cell granules stain purple in color due to the presence of heparin and histamine. For this reason, purple stain was recognized as a positive staining for mast cells. For immunofluorescence study, sections were dewaxed with xylene and hydrated by graded alcohols. Antigen retrieval was performed by incubating the dewaxed and hydrated sections with 10 mM sodium citrate buffer, pH 6, at 95 °C for 10 min. The sections were washed three times with PBST (0.05% Tween-20 in phosphate buffered saline). The tissue sections were then blocked by incubation with 2% bovine serum albumin for 1 h at room temperature. The tissue sections were incubated with anti-chicken DRA primary antibody (custom antibody, Invitrogen Life Technologies) (diluted 1:100) for 1 h at room temperature. Excess primary antibody was removed with PBST three times followed by incubation with secondary antibody Alexa Fluor 488 goat anti-chicken (Cat No. A11039; Invitrogen Molecular Probes, Carlsbad, CA, USA) (diluted 1:500) for 1 h at room temperature. Excess secondary antibody was removed with PBST three times, and the section was mounted with Fluoroshield mounting medium with 4,9,6-diamidino-2-phenylindole (DAPI) for nucleus staining (ab104139, Abcam, Cambridge, MA, USA). Images were captured with an EVOS FL Cell imaging system with the same exposure time and magnification in all the conditions. Omission of primary antibodies was used as a control. Quantification of florescence intensity was determined by Image J Software (version 1.52n, National Institute of Health, Bethesda, MD). Equal areas from each of the villus brush border membranes were selected by rectangular tools in image j to measure the raw integrated density. Relative fluorescence unit (RFU) was calculated from the raw integrated density.

### 2.11. Protein Assay

Total proteins were quantified by Lowry’s method using the DCTM protein assay kit (Bio-Rad, Berkeley, CA, USA) for all the uptake and molecular studies described in this study.

### 2.12. Statistical Analysis

Results are shown as means ± standard error of mean (SEM), calculated with GraphPad Prism 7 (San Diego, CA, USA) software. All the uptake experiments were performed in quadruplicate. The “n” number indicates experiments performed with cells isolated from different animals. Student’s *t*-test was performed for statistical analysis with GraphPad Prism 7 software and *p* < 0.01 was considered significant.

## 3. Results

### 3.1. Effect of Ketotifen on Mast Cell Degranulation in Chronically Inflamed SAMP1 Mice

**Figure 1:**
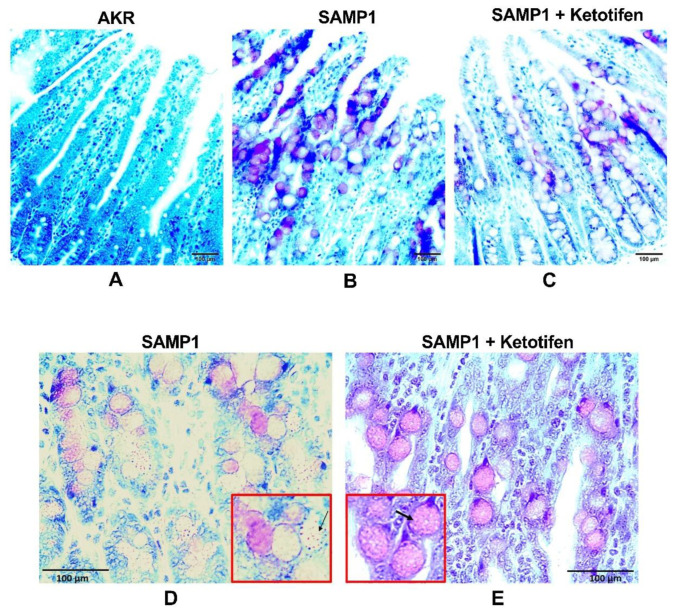
Effect of ketotifen on mast cell degranulation. Ileum of AKR (**A**), SAMP1 (**B**) and SAMP1 mice treated with ketotifen (**C**) were stained by toluidine blue. A marked increase in number of mast cells was observed by toluidine blue staining in SAMP1 mice (**B**,**C**). Original magnification 10×. Degranulation status of mast cell in SAMP1 (**D**) and SAMP1 mice treated with ketotifen (**E**) were observed in the presence of metachromatic blasts. Original magnification 40×. (D) In SAMP1 mice, degranulated mast cells were characterized by less intense metachromasia with free granules within the cytoplasm and outside of the cell border (indicated by thin black arrow). (E) In vivo treatment of SAMP1 mice with ketotifen prevented the degranulation of mast cells, which were round or oval with uniform color and intact cell membranes (indicated by bold black arrow).

As shown in Figure 1A, mast cells are rarely found in AKR mice ileum. However, in SAMP1 mice ileum, many mast cells were scattered throughout the lamina propria in between the villus and close to the crypts (Figure 1B,C). To observe the mast cell degranulation, 40× image was captured. In SAMP1 mice, degranulated mast cells exhibited less intense metachromasia, unclear or irregular cell membranes and had free granules within the cytoplasm and outside of the cell border (Figure 1D). In vivo treatment of SAMP1 mice with ketotifen reduced the degranulation of mast cells, which appeared round or oval with uniform color and intact cell membranes (Figure 1E). This result indicates that in chronic ileitis, there is a marked increase in the number of mast cells accompanied by elevated degranulation, whereas, in vivo ketotifen treatment stabilized the mast cells to prevent its degranulation during inflammation.

### 3.2. Effect of Ketotifen on Mast Cell β-Hexosaminidase in Intestinal Mucosa

**Figure 2:**
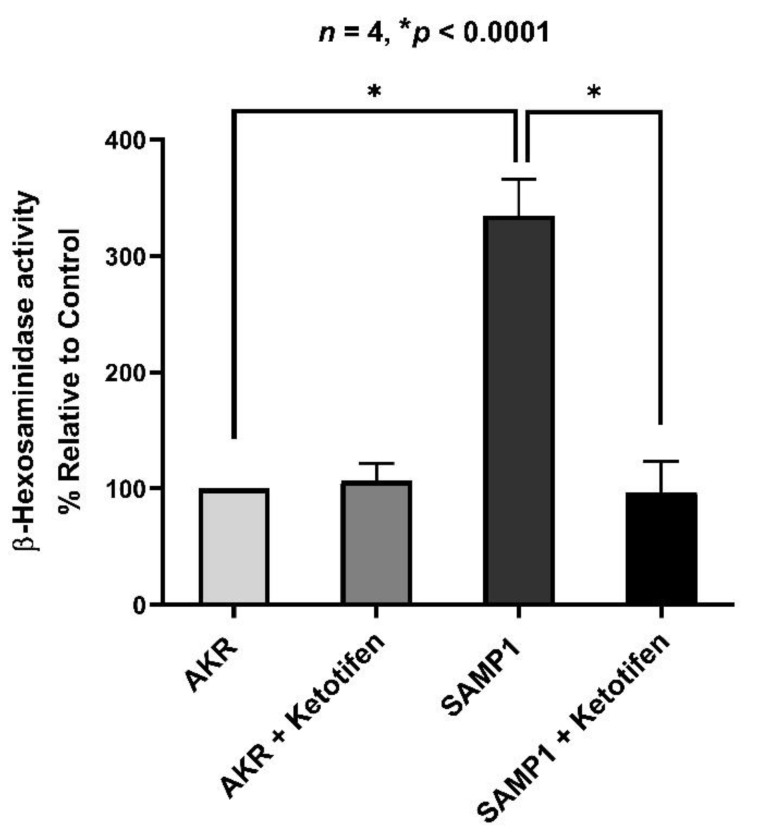
Effect of ketotifen on β-hexosaminidase activity in small intestinal villus cells. β-hexosaminidase activity was significantly increased in intestinal villus cells isolated from the chronically inflamed SAMP1 mice when compared to AKR control mice. In vivo treatment with ketotifen in SAMP1 mice restored β-hexosaminidase activity at normal levels while having no effect in the ketotifen treated AKR mice.

Mast cells contain a large number of enzymes that are known to be involved in several inflammatory pathways. β-hexosaminidase is a glycolytic enzyme found in abundance in mast cells. β-hexosaminidase is commonly used as a biomarker of mast cell degranulation during inflammation. In the present study, β-hexosaminidase activity was significantly increased in villus cells isolated from the SAMP1 intestine compared to that of AKR mice indicating enhanced degranulation of mast cells in SAMP1 mice. However, this increased β-hexosaminidase activity in SAMP1 mice was reversed back to the normal levels by in vivo ketotifen treatment of SAMP1 mice, which indicates that mast cell degranulation was prevented by ketotifen (Figure 2). There was no change in β-hexosaminidase activity levels in intestinal villus cells isolated from treated and untreated AKR mice.

### 3.3. Effect of Ketotifen on Cl^−^/HCO_3_^−^ Exchange in the BBMV of Ileal Villus Cell

**Figure 3:**
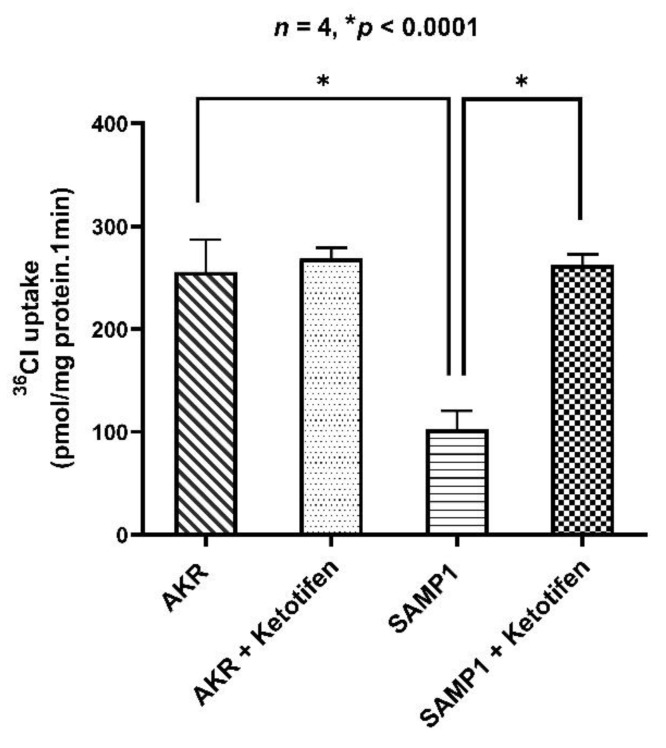
Effect of ketotifen on Cl^−^/HCO_3_^−^ exchange in BBMV. Cl^−^/HCO_3_^−^ exchange was significantly decreased in ileal villus cell BBMV from SAMP1 mice compared to that of AKR mice. In vivo ketotifen treatment reversed the decreased BBM Cl^−^/HCO_3_^−^ exchange activity in SAMP1 mice to normal levels. Cl^−^/HCO_3_^−^ exchange activity remained unchanged in AKR mice treated with ketotifen when compared to that of untreated AKR control mice.

Cl^−^/HCO_3_^−^ exchange, defined as HCO_3_^−^-dependent and DIDS sensitive Cl uptake, was significantly reduced in villus cell BBMV from SAMP1 mice compared to AKR mice. In order to determine whether mast cell mediators are responsible for the decrease in Cl^−^/HCO_3_^−^ exchange in the BBM, SAMP1 mice were treated in vivo with ketotifen. Ketotifen treatment of SAMP1 mice resulted in a significant reversal of Cl^−^/HCO_3_^−^ exchange in villus cell BBMV (279 ± 15 pmol/mg protein/min in AKR; 102 ± 8 pmol/mg protein/min in SAMP1; 268 ± 5 pmol/mg protein/min in AKR + ketotifen; 268 ± 5 pmol/mg protein/min in SAMP1 + ketotifen), but had no effect in AKR mice (Figure 3). These results indicate that Cl^−^/HCO_3_^−^ exchange diminished during chronic inflammation was reversed by in vivo ketotifen treatment by mast cell stabilization in ileal villus cell.

### 3.4. Na/H Exchanger (NHE3) Activity in the BBMV of Ileal Villus Cell

**Figure 4:**
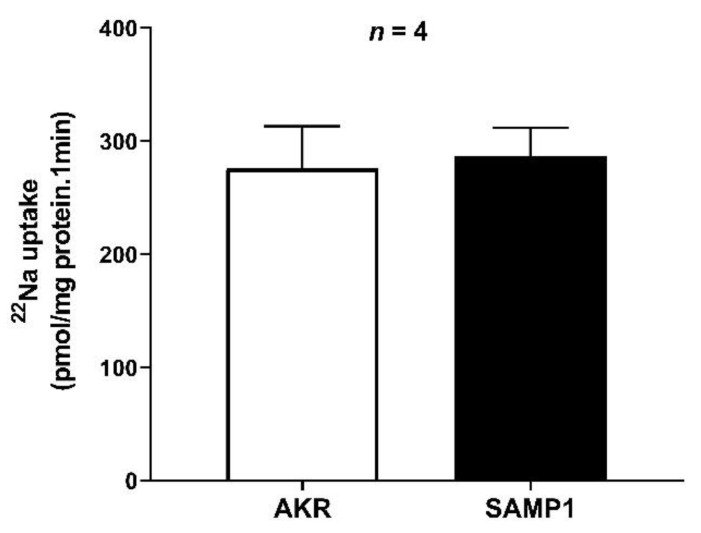
Na/H exchanger (NHE3) activity in ileal villus cells BBMV. pH dependent and amiloride sensitive ^22^Na uptake was measured as NHE3 activity, which remained unchanged in BBMVs of intestinal villus cells from SAMP1 mice compared with AKR control mice.

Na/H exchange was unaffected in the chronically inflamed intestine of SAMP1 mice compared to that of AKR control mice (276 ± 20 pmol/mg protein/min in AKR; 287 ± 18 in SAMP1) (Figure 4). This result indicates that the inhibition of coupled NaCl absorption during chronic ileitis is secondary to the inhibition of Cl^−^/HCO_3_^−^, but not Na/H exchange.

### 3.5. Kinetic Studies for Cl^−^/HCO_3_^−^ Exchange

**Figure 5:**
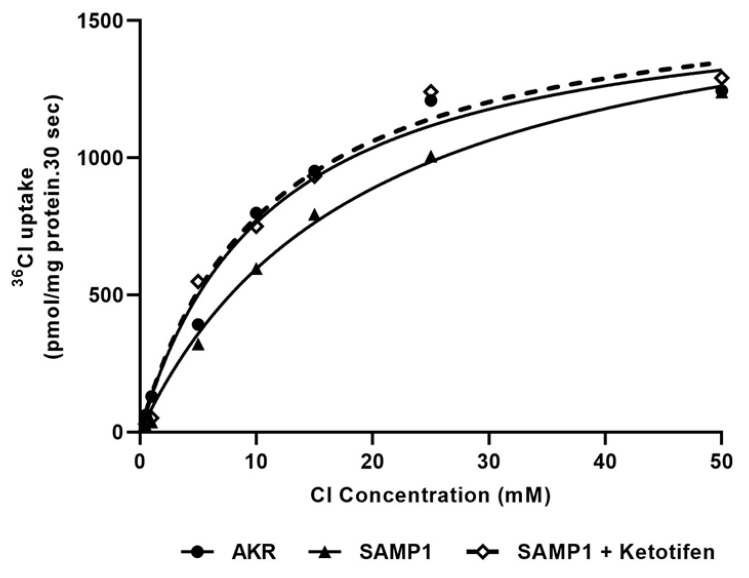
Kinetics of Cl^−^/HCO_3_^−^ exchange in intact intestinal villus cells from AKR, SAMP1 and ketotifen treated SAMP1 mice. HCO_3_^−^-dependent and DIDS-sensitive ^36^Cl uptake is shown as a function of varying concentrations of extra vesicular Cl^−^ at 30 s. As the concentration of extra-vesicular Cl^−^ was increased, ^36^Cl uptake was stimulated and subsequently became saturated in all conditions. Analysis of the data yielded kinetic parameters. In villus cells isolated from SAMP1 mice the Cl^−^/HCO_3_^−^ exchange was inhibited by altering the affinity of the transporter for Cl^−^ and ketotifen treatment significantly reversed the inhibited Cl^−^/HCO_3_^−^ exchange in SAMP1 villus cell by restoring the affinity of the transporter (K_m_) without an alteration in the maximal rate of uptake (V_max_) of Cl.

To determine the mechanisms of reversal of the Cl^−^/HCO_3_^−^ exchange activity by ketotifen in inflamed villus cells, we performed intact villus cell kinetic studies. Figure 5 shows ^36^Cl uptake in intact villus cell as a function of varying concentrations of extra-vesicular Cl. As the concentration of extra-vesicular Cl was increased, ^36^Cl uptake was stimulated and subsequently became saturated in all conditions. Kinetic parameters demonstrated that in the intact intestinal villus cells isolated from SAMP1 mice inhibition of Cl^−^/HCO_3_^−^ exchange activity occurs as a result of an increase in the K_m_, and in vivo ketotifen treatment reversed this change (K_m_ was 11.14 ± 0.5 mM in AKR, 19.44 ± 0.3 mM in SAMP1, 11.01 ± 0.3 mM in SAMP1 + Ketotifen; *n* = 4). The maximal rate of uptake (V_max_) was unaffected in all conditions (V_max_ was 1.62 ± 0.04 nmol/mg protein/30 s in AKR, 1.75 ± 0.03 in SAMP1, 1.65 ± 0.05 in SAMP1 + Ketotifen; *n* = 4). These data indicate that the mechanism of reversal of inhibition of Cl^−^/HCO_3_^−^ exchange by ketotifen during chronic intestinal inflammation was secondary to a restoration in the affinity for Cl^−^ rather than an alteration in the number of intestinal villus cell Cl^−^/HCO_3_^−^ exchangers.

### 3.6. DRA mRNA Expression by RT-qPCR

**Figure 6:**
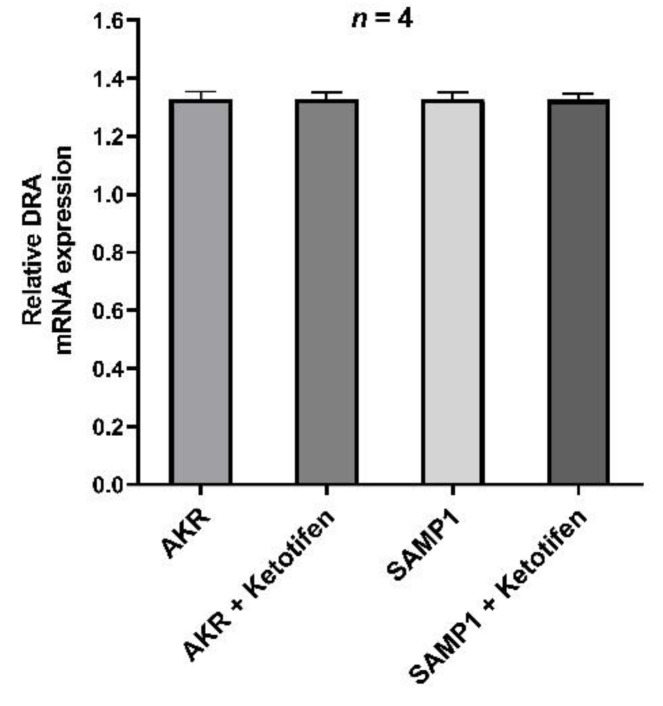
RT-qPCR analysis of DRA mRNA expression in intestinal villus cells isolated from ketotifen treated and untreated AKR, and SAMP1 mice. DRA mRNA expression remained unchanged in all experimental samples. The data obtained with β-actin was used to normalize the expression levels of DRA between individual samples.

In order to elucidate the molecular mechanism of mast cell regulated inhibition of the major Cl^−^/HCO_3_^−^ exchanger such as DRA, we determined its mRNA expression in villus cells by RT-qPCR. The results showed that DRA specific mRNA expression remained unchanged in all experimental samples (Figure 6). This data has consistency with kinetic parameters that demonstrate that the mechanism of ketotifen mediated reversal of inhibition of Cl^−^/HCO_3_^−^ exchanger activity in the villus cells isolated from the chronically inflamed intestine was secondary to the restoration of the affinity of the co-transporter for its substrate without a change in the transporter numbers.

### 3.7. Western Blot Studies for DRA

**Figure 7:**
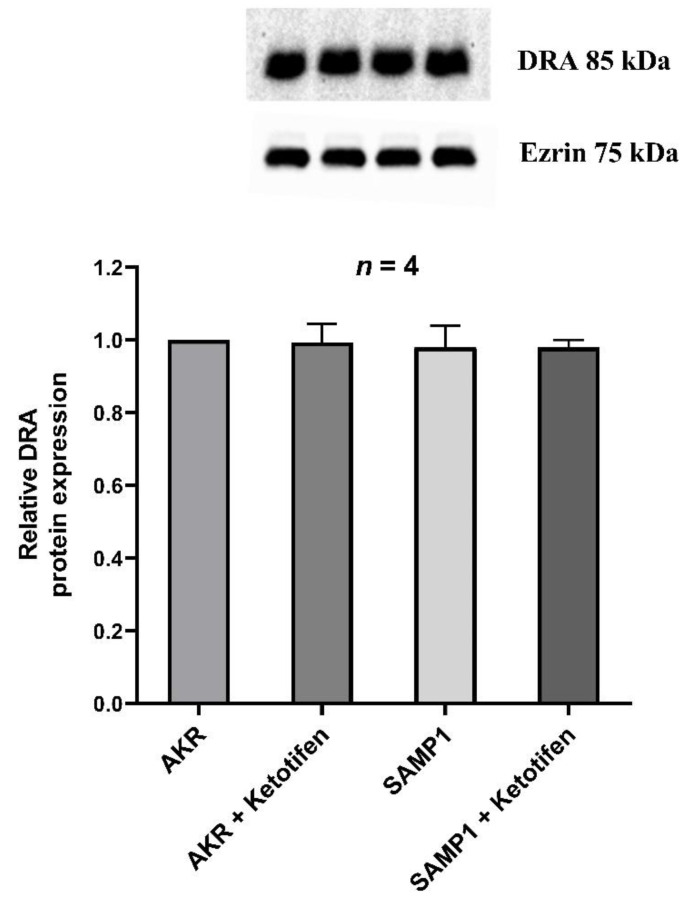
Effect of ketotifen on villus cell BBM DRA protein expression during chronic ileitis. Western blot analysis showed that villus cell BBM DRA protein levels remained unaltered in all experimental conditions (a representative blot in the top panel). Densitometric analysis as shown in the bottom panel confirmed these findings.

Since the mRNA level may not necessarily correlate with the immunoreactive protein in the BBM, we quantitated DRA specific immunoreactive protein. There was no significant difference in the immunoreactive levels of DRA in any of the conditions tested (Figure 7). Densitometric quantitation confirmed these observations (Figure 7). These results, in conjunction with kinetic parameters and RT-qPCR data suggested that the mechanism of inhibition of Cl^−^/HCO_3_^−^ exchanger activity by the mast cell degranulation during chronic intestinal inflammation was secondary to a decrease in the affinity of the transporter for Cl^−^ without a change in the transporter numbers.

### 3.8. Immunofluorescence Studies of DRA

**Figure 8:**
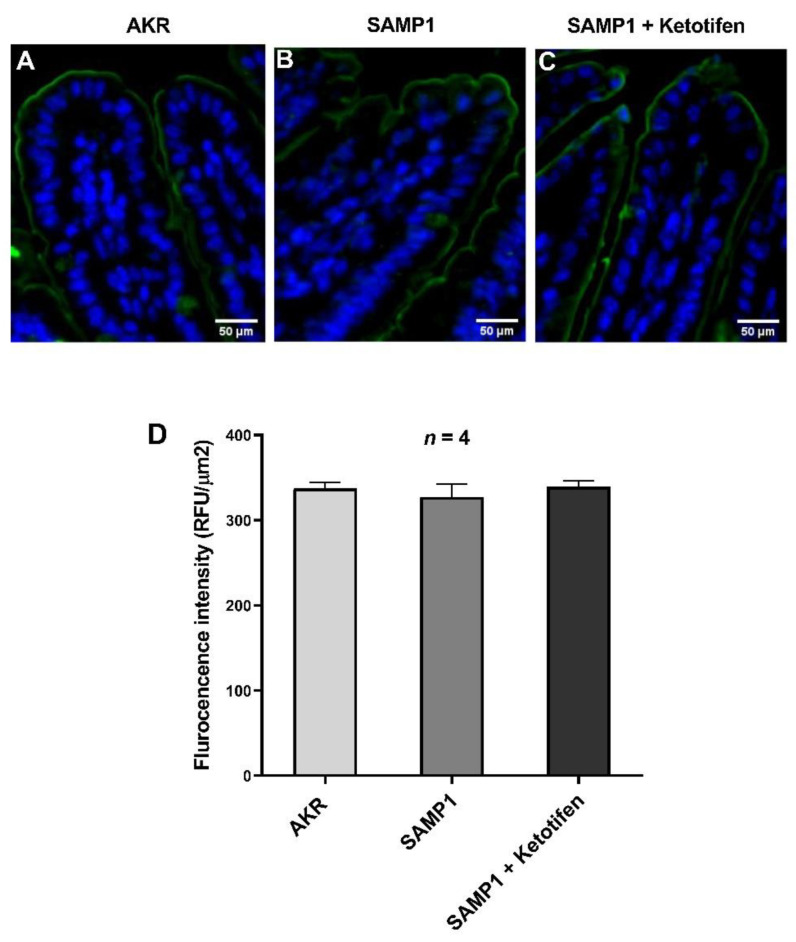
Immunofluorescence of DRA in small intestine of AKR, SAMP1 and ketotifen treated SAMP1 mice. Images captured at 20× magnification. (**A**) DRA in AKR mouse intestine demonstrating its abundance in the BBM of villus cells lining the villus. (**B**) In SAMP1 mouse intestine demonstrating DRA in the BBM of villus cells lining the villus remained unaltered during chronic ileitis compared with AKR villus cells. (**C**) DRA in ketotifen treated SAMP1 mouse intestine demonstrating that DRA expression is unchanged in the BBM of villus cells. (**D**) Quantitation analysis showed that DRA expression was not altered in small intestine of AKR, SAMP1 and ketotifen treated SAMP1 mice.

In order to localize DRA in the mice ileum and to further confirm if it is affected by chronic intestinal inflammation in SAMP1 mice, immunofluorescence was performed along with AKR control mice. Figure 8 demonstrates that DRA is present in the BBM of mice small intestinal villus cells. Further, DRA protein expression was unaltered during chronic intestinal inflammation in SAMP1 mice compared to that of AKR mice. In SAMP1 mice treated with ketotifen DRA protein expression also remained unchanged. Altogether, immunofluorescence results along with molecular studies and kinetic parameters demonstrate that Cl^−^/HCO_3_^−^ exchange inhibition during chronic inflammation is not secondary to a decrease in protein expression.

## 4. Discussion

Mast cells in the gastrointestinal mucosa are known to be involved in a number of physiological and pathophysiological functions in the normal and chronically inflamed intestine. Its primary physiological function is to maintain tissue homeostasis and guard the intestinal barrier as it is the pivotal site for the initiation and development of immune and infectious diseases [33]. In pathophysiological conditions such as in IBD or in infectious bowel disorders, mast cell degranulation has been linked to epithelial barrier dysfunction and development and progression of inflammation by the activation of adaptive immune response mechanisms [33,34]. Specifically, degranulation of intestinal mast cells releases a variety of mast cell mediators including histamine, serotonin, proteases, and proteoglycans, as well as newly synthesized factors such as cytokines, growth factors, and free radicals [34]. Mast cells and their mediators are known to play a vital role in the onset and progression of IBD [35,36,37,38,39,40]. However, only very few studies have been documented in the literature regarding the involvement of mast cells in the regulation of intestinal ion transport [24,41,42,43,44].

In the present study, infiltrated mast cell numbers were shown to be significantly increased in the small intestine of the SAMP1 mouse model of chronic spontaneous ileitis compared to control AKR mice. Moreover, β-hexosaminidase levels, which serve as an indicator of mast cell degranulation [45], were significantly increased in the intestinal extracts from SAMP1 mice compared to AKR mice suggesting the possible role of mast cell mediators not only in the progression of inflammation in SAMP1 mice but also in the downregulation of chloride absorption in the BBM of villus cells from SAMP1 mice. Further experimental data derived in this study from ketotifen treated animals confirmed the involvement of mast cell mediators in chloride malabsorption in SAMP1 mice. In this study, we used ketotifen as the mast cell stabilizing agent, since we had successfully used this agent previously in our rabbit model of chronic intestinal inflammation to demonstrate the effect of mast cell stabilization on intestinal nutrient transport regulation [46]. In SAMPI mice, treatment with ketotifen reversed the β-hexosaminidase levels to near normal levels with concurrent restoration of villus cell BBM Cl:HCO_3_ exchange activity to normal levels, suggesting that mast cell mediators were likely involved in the downregulation of chloride absorption in chronic intestinal inflammation. Though the identity of the specific mast cell mediator that might regulate chloride malabsorption in SAMP1 mice is yet to be deciphered, previously reported in vivo studies have indeed demonstrated that mast cell mediators, specifically histamine and serotonin, regulate small intestinal ion transport [29,47].

As demonstrated in a previous study [27] and in this current study, downregulation of Cl:HCO_3_ exchange mediated by DRA in the BBM was found to be secondary to the altered affinity of DRA for its substrate chloride rather than an alteration in its maximal velocity of chloride absorption. Western blot confirmed this finding with unaltered expression of DRA in the BBM of villus cells. This altered affinity of DRA in the villus BBM of SAMP1 mice was found to be restored to normal by ketotifen treatment in the present study. RT-qPCR data analyses revealed that DRA mRNA expression remained unchanged in all experimental conditions confirming that altered transcription was unlikely to be the mechanism of altered DRA expression in the different experimental conditions of this study. Likewise, Western blot analysis of DRA BBM protein expression corroborated the kinetic studies and RT-qPCR data establishing that ketotifen treatment restored DRA activity exactly by the same mechanism that downregulated its activity in SAMP1 mice. This finding is comparable to a previous study where inhibition of inducible nitric oxide by L-NIL treatment reversed DRA activity by the same mechanism that was initially responsible for its downregulation in the SAMP1 mouse model of chronic ileitis [27]. Moreover, in a related study, it was further established that altered affinity of DRA leading to its downregulation in chronic intestinal inflammation was due to increased phosphorylation in its serine and threonine residues, which was also restored back to its normal levels by L-NIL treatment [28]. Contrary to our molecular findings in the inflamed small intestine of SAMP1 mice, several studies conducted in the mice model of Dextran sulfate sodium (DSS) induced colitis and Rag2^−^/^−^ mice have demonstrated that DRA downregulation in colon is due to a decrease in its mRNA and protein expression [48,49,50,51]. These contrary findings indicate that the molecular mechanism of downregulation of DRA in intestinal inflammation is unique to the small and large intestines.

Both DRA and PAT1 have been traditionally known to be the predominant chloride absorptive mechanisms in the BBM of villus cells in the mammalian gastrointestinal tract. However, the inevitable significance of DRA over PAT1 as the mediator of diarrheal phenotype is evident from several studies found in the literature. Most importantly, congenital chloride diarrhea in humans, which is an autosomal recessive disorder due to mutations in the DRA gene *slc26a3*, is characterized by extensive diarrhea, loss of chloride, and metabolic alkalosis [52,53,54,55,56]. Similarly, in DRA knockout mice, which mimics the congenital chloride diarrhea, severe chloride losing diarrhea resulting in serum electrolyte imbalance, metabolic alkalosis, and growth retardation is known to occur [57]. However, *slc26a6* (PAT1) deficient mice do not manifest diarrheal phenotype indicating the insubstantial importance in chloride malabsorption [58]. This explains the significant observation in our previous study in SAMP1 mice, where only DRA was affected at the level of its protein in the BBM of villus cells by altered phosphorylation levels, whereas PAT1 remained unaffected [28], establishing the singular role of DRA in downregulation of chloride absorption in IBD.

In various experimental in vivo models of IBD, such as in DSS and Trinitrobenzenesulfonic (TNBS) colitis rat models and in IL-10 deficient mice model, increased mast cell proliferation and degranulation has been shown to play a critical role in disease progression [59,60,61,62,63]. In human IBD patients, in addition to showing increased mast cells in the lamina propria and submucosa, evidences of degranulation of mast cells in terms of increased expression of TNFα, IL6, substance P and elevated histamine, prostaglandins, leukotrienes, and tryptase levels have been demonstrated [37,64,65]. It is likely that one of the abovementioned mast cell mediators is involved in the downregulation of DRA in SAMP1 mice model of chronic ileitis. In a study conducted in a rabbit model of chronic enteritis it was shown that the cyclooxygenase pathway rather than the lipooxygenase pathway is involved in the downregulation of DRA in chronic small intestinal inflammation [19]. There again, the downregulation of DRA was due to altered affinity of the BBM transporter for its substrate. In an interesting study published in 1992 in the TNBS model of colitis, ketotifen treatment was found to significantly decrease macroscopic damage to the colon accompanied by a decrease in prostaglandin E2, thromboxane B2, leukotriene B4 and C4 generation, and nitric oxide synthase activity [66]. Summing all this literature, a prostaglandin released by the mast cell in response to chronic inflammation, may be responsible for altered downregulation of DRA in SAMP1 mice as seen in the present study. Ongoing studies in SAMP1 mice and in SAMP1 mice derived organoids will establish the identity of the specific immune inflammatory mediator and the functional and molecular mechanisms responsible for the downregulation of DRA in SAMP1 mice.

The treatment drugs used currently for the treatment of IBD are known to affect mast cell activities. For example, corticosteroids are well known to reduce mast cell numbers in human IBD [67]. Also, 5-aminosalicylic acid has been shown to inhibit histamine and prostaglandin D2 release from intestinal mast cells [23,68]. Interestingly, a handful of studies performed in the 1990s investigated the therapeutic potential of ketotifen for the treatment of human IBD, which showed limited advantageous outcomes [69,70]. Based on the current study, the therapeutic potential of mast cell stabilizers in the treatment of IBD, most importantly in the treatment of chloride malabsorption in IBD diarrhea, needs to be reevaluated as this might help to overcome some of the adverse effects seen by patients using corticosteroids, which is the current mainstay of treatment for IBD patients.

In conclusion, DRA mediated downregulation of chloride malabsorption was significantly restored by the mast cell stabilizer ketotifen in the SAMP1 model of spontaneous chronic ileitis. The mechanism of restoration of DRA activity was secondary to the restoration of its altered affinity. Therefore, mast cell mediator/s are likely involved in the downregulation of chloride absorption in chronic small intestinal inflammation.

## Data Availability

Not applicable.
